# O093: Hand rub dance: an edutainment tool to remember the steps of hand hygiene procedure

**DOI:** 10.1186/2047-2994-2-S1-O93

**Published:** 2013-06-20

**Authors:** A-G Venier, C Bervas, A Chasseuil, P Parneix

**Affiliations:** 1 CCLIN Sud-Ouest, Bordeaux, France

## Introduction

The need for, and benefits of, hand hygiene technique training have been identified. However, it usually requires actions which are often logistically challenging and time-consuming. For the 2012 French hand hygiene day, which was in November due to political schedule, an edutainment concept was created: the hand rub dance.

## Objective

To educate many people in a short time to hand hygiene technique.

## Methods

It was suggested to facilities of South-western France to perform hand rub dance in November 2012: principle was to teach the choreography to volunteers, to film the dancers and send videos to create a compilation. The choreography of the hand rub dance was based on the different steps of hand hygiene (palm to palm, palm over dorsum, fingers interlaced, backs of fingers, thumbs, fingertips and wrists). Tool, also available in an English version, included a methods guide with practical and technical information, a didactic video, a training video and a royalty-free original music (http://danse-du-sha.fr/). It was intended for healthcare facilities and educational institutions and could address a variety of audiences such as healthcare workers, patients, students, executive managers or visitors.

## Results

The training video was viewed online 5,000 times. Participation rate was 11% with 88 videos from 51 healthcare facilities and 4 educational institutions from the whole South-western France. Participants were mostly public hospitals (24%), rehabilitation centres (16 %), nursing facilities (14 %) and private hospitals (9 %). More than 1,400 people were filmed (median: 20 people per video, 170 maximum). Healthcare workers appeared in 86 % of videos. Patients and visitors danced in 12 % of videos. Participants noticed a rapid memorization by the targeted audience of the seven steps of hand hygiene. The compilation was set online and viewed 3,000 times since its release in late February 2013.

## Conclusion

Hand rub dance is a complementary tool to promote hand hygiene; 2012 experience in South-western France motivated and gathered a large and eclectic public on hand hygiene theme and the compilation acted as a complementary tool for hand hygiene promotion. Hand rub dance will be suggested to national and international facilities for WHO hand hygiene day in May 2013.

## Disclosure of interest

None declared.

